# Cervical stump leiomyomata after supracervical hysterectomy; a case report with review of literature

**DOI:** 10.1186/s12905-024-03326-2

**Published:** 2024-09-10

**Authors:** Ahmed Shoukry, Mahmoud Yousri

**Affiliations:** Department of Obstetrics and Gynecology, Faculty of Medicine, Alexandria University, Alexandria, Egypt

**Keywords:** Subtotal, Supracervical, Hysterectomy, Stump, Fibroids

## Abstract

**Background:**

Despite being a highly debated issue, subtotal or supracervical hysterectomy (SCH) is still considered a safe and effective treatment for women with benign gynecological lesions. Benign and malignant cervical diseases have been reported after SCH, with fibroids being the most frequently diagnosed lesions in the excised cervical stump. Recurrence of cervical disease after SCH usually presents with vaginal bleeding, pelvic mass, or abdominal pain; moreover, it may necessitate reoperation and resection of the cervical stump or trachelectomy. Trachelectomy is known to be a difficult surgical procedure that may be associated with significant intra- and post-operative morbidity.

**Case presentation:**

We presented here a case of a 41-year-old nulliparous woman with a pelvic mass related to the cervical stump presented 2 years after subtotal hysterectomy, performed due to interactable abnormal uterine bleeding, which was attributed to a multiple fibroid uterus. Six years ago, she complained of pelvic pain, excessive vaginal discharge, and spotting. A transvaginal sonography and magnetic resonance imaging with contrast were performed, which revealed a 10.2 × 7.6 × 6.5 cm heterogeneous pelvic mass with irregular borders and marked vascularity on color Doppler. Surgical exploration and resection of the mass with cervical stump excision were performed. Histopathology confirmed the diagnosis of cervical stump multiple benign leiomyomata with no atypical features.

**Conclusion:**

Recurrence or De novo development of leiomyomata and other cervical lesions might occur after supracervical or subtotal hysterectomy; thus, thorough pre-operative counseling for women requesting a SCH regarding the pros and cons of the procedure compared with total hysterectomy should be optimized. Meticulous follow-up, including the continuation of routine cervical cytological smears, is mandatory for patients with a retained cervix.

## Background

Hysterectomy is the most frequently performed surgical procedure for women with symptomatic uterine fibroids [1, 2].

Preservation of the cervix uteri during a hysterectomy is a highly debated issue. Some of the potential benefits of supracervical hysterectomy (SCH) that proponents often mention are reduced blood loss, a shorter hospital stay, fewer intraoperative risks, fewer urinary complications, and better pelvic support [3–5]. Although postoperative sexual function is presumed to be more satisfactory after SCH [6], it was not found to be superior to a total hysterectomy (TH) [7]. On the contrary, some problems have been often described with SCH, such as cyclic bleeding, pelvic pain, and a higher incidence of cervical disease [8]. Among the commonest clinical indications for surgical excision of the cervical stump following supracervical hysterectomy are pelvic masses and vaginal bleeding [9]. It has been observed that fibroids are the most common (in approximately 35%) pathology found in the excised cervical stump [10].

We report a case of large stump fibroids after a subtotal abdominal hysterectomy for multiple fibroid uterus.

## Case report

### Chief complaints

A 41-year-old nulliparous woman presented to the gynecology outpatient clinic of El-Shatby University Hospital, Alexandria, Egypt, with pelvic pain and excessive vaginal discharge with occasional vaginal spotting.

### History of the current illness

The patient had chronic dull, aching pelvic pain with a heaviness sensation. She also described an increased amount of brownish vaginal discharge. Her symptoms started in 2018, and she reported that the condition became noticeably worse six months ago with frequent episodes of vaginal bleeding, so she sought gynecological consultation.

### History of the past illness

She gave a history of four abdominal myomectomies for the excision of multiple fibroids while she was seeking fertility; the last myomectomy was performed in 2014. After that, she complained of intractable vaginal bleeding. A multiple fibroid uterus was diagnosed, so she underwent a supracervical abdominal hysterectomy with preservation of both ovaries in 2016. All previous surgeries were performed outside our hospital through midline vertical incisions.

Hysterectomy specimen examination revealed a 2500-gm uterus with multiple interstitial and subserous leiomyomata ranging from 2 to 9.5 cm. Histopathological examination confirmed the diagnosis of typical leiomyoma pathological features with no detected atypical or mitotic figures.

### Medical, personal, and family history

She gave history of two failed IVF trials after her first and third myomectomy. Her medical history is irrelevant except for mild bronchial asthma controlled with periodic beta-agonist inhalers. She did not have a history of smoking or drinking alcohol. She did not undergo any pap smears either before or after the supracervical hysterectomy. She denies any follow-up visits to her gynecologist after the hysterectomy.

### Physical examination

On examination, her abdomen was lax and non-tender, with no palpable masses. The previous midline incisions were noticed with no palpable hernias. A bimanual examination revealed a firm, non-tender pelvic mass that has limited mobility in conjugation with movement of the cervix. A speculum examination showed a normal-looking cervix with a well-estrogenized vagina.

### Imaging examinations

A transvaginal ultrasound revealed a 10.2 × 7.6 × 6.5 cm pelvic mass with irregular borders and heterogeneous echotexture with no external shadowing. There were internal cystic areas resembling the picture of degenerated fibroid. The mass had marked vascularity on the color Doppler, especially in the peripheral regions. The mass was seen encroaching on the right adnexa, with the normal appearance of the left ovary.

**Fig. 1:**
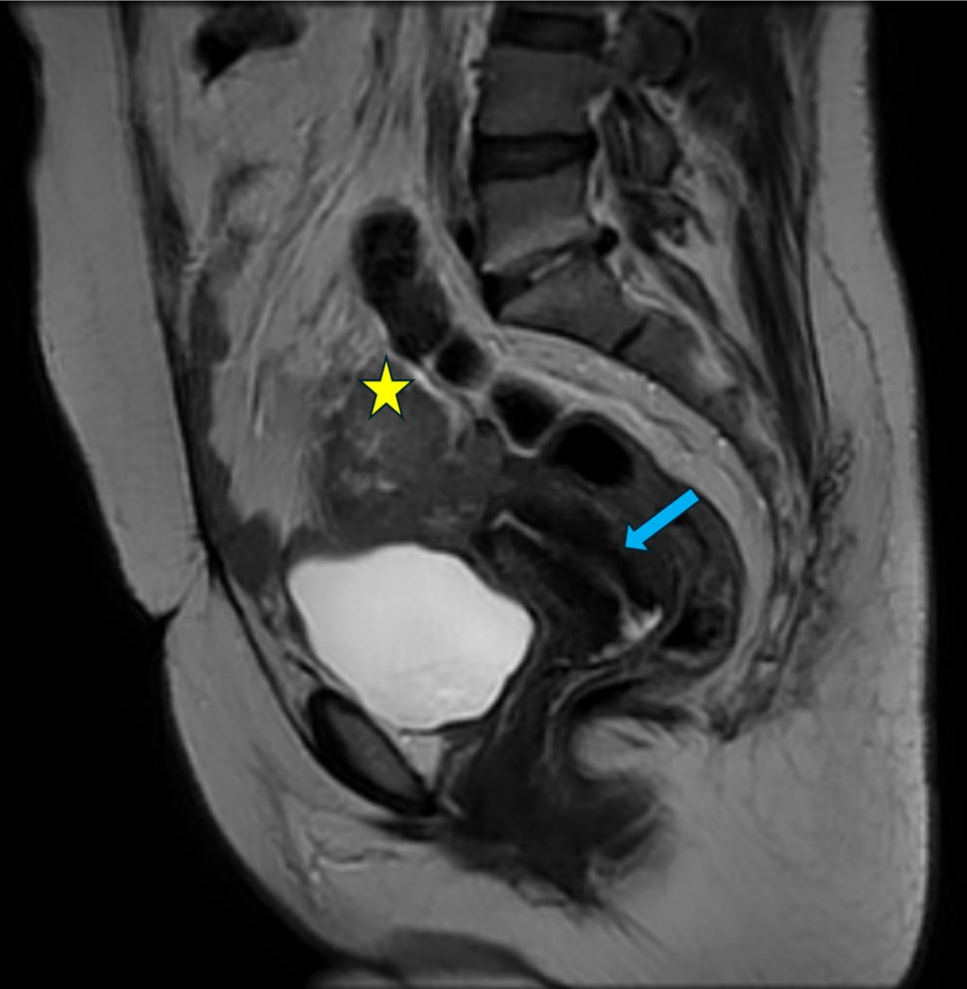
Sagittal T2W image showing the remaining cervical stump with its endometrial lining (blue arrow) and a heterogeneous solid lesion (yellow star) above the cervix with its MRI signal matching with fibroid

**Fig. 2:**
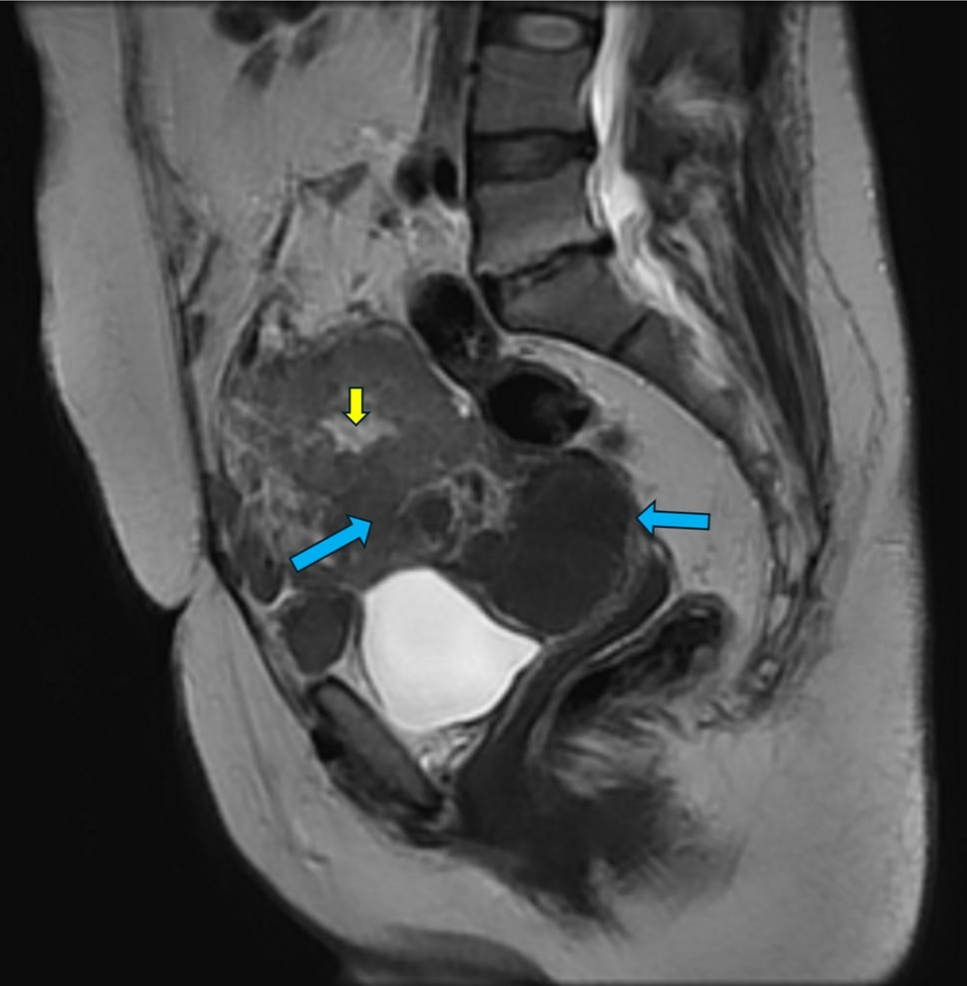
Sagittal T2W image showing heterogeneous lobulated mainly hypo-intense solid (blue arrows) lesion showing areas of cystic changes (small yellow arrows) consistent with degenerated cervical leiomyomata

The mass looks suspicious for malignancy, so confirmatory magnetic resonance imaging (MRI) with IV gadolinium contrast was performed, as demonstrated in Figs. 1 and 2. The heterogeneous T2 lobulated mixed solid and cystic lesion showed a mainly hypo-intense signal in the T2 and T1 weighted images, epicentered upon the cervical stump and engulfing the right adnexa.

The patient was counseled for exploratory laparotomy and resection of the mass with the cervix and both ovaries. A complete preoperative workup was performed, including a chest x-ray; all investigations were within normal range.

### Surgical details

An extended lower midline incision was performed to optimize the exposure of the entire abdominal cavity and the pelvis. Upon entry of the abdomen, a large pelvi-abdominal mass was found, which was entirely covered by densely adherent bowel loops posteriorly and an exceptionally adherent urinary bladder anteriorly.

The lateral approach, through opening the retroperitoneal avascular pelvic spaces, was used to properly identify the borders of the mass and to secure the important adjacent structures, namely the ureters, iliac vessels, and recto sigmoid.

Creation of the para-vesical and para-rectal spaces was performed bilaterally, which facilitated the identification of the ureters and iliac vessels, followed by ureterolysis and lateralization of both ureters. Dissection of the densely adherent recto-sigmoid off the back of the mass with development of the rectovaginal space and identification of uterosacral ligaments was done.

**Fig. 3:**
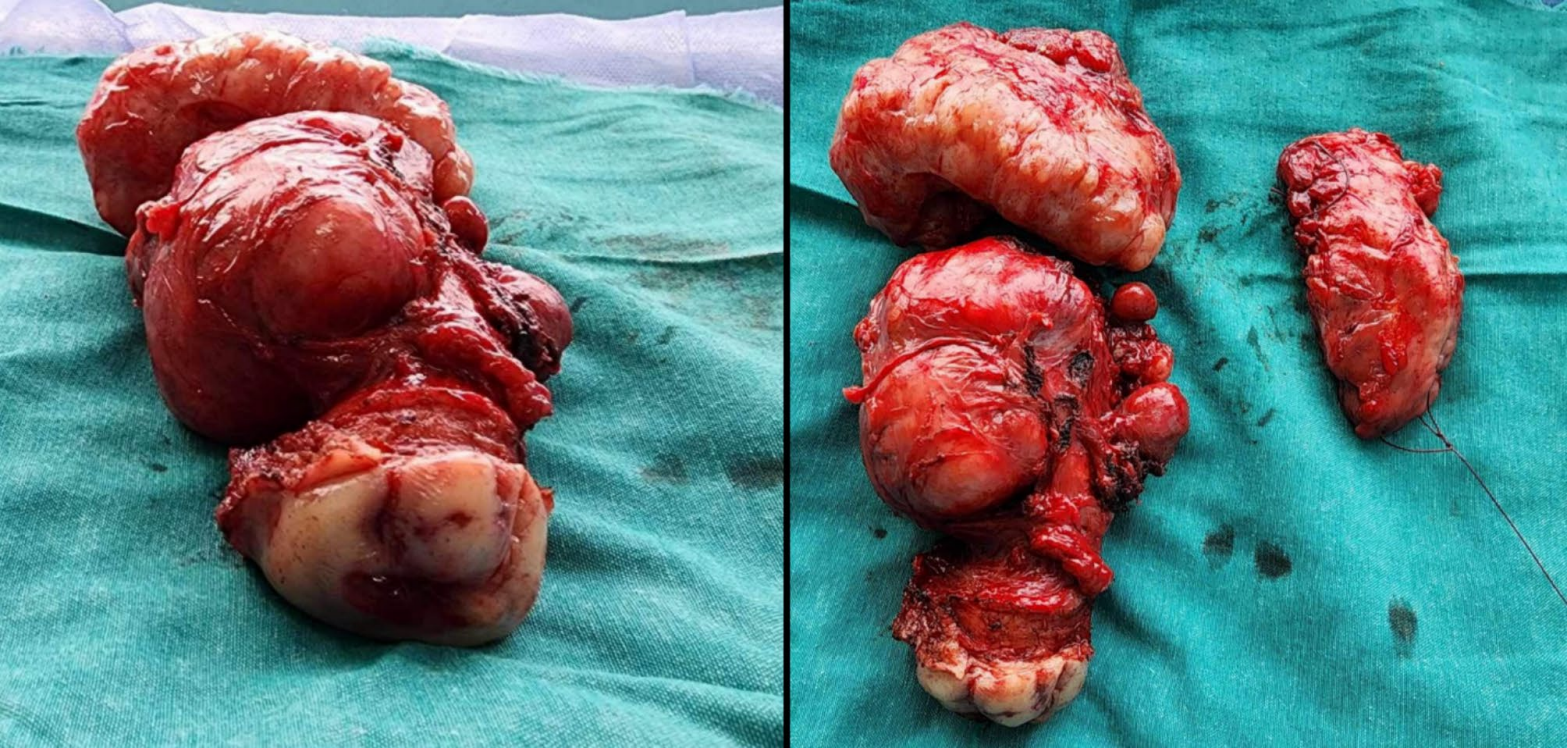
The surgical specimen showing multiple leiomyomata and the excised cervical stump

A decision was made to excise the fibroids from the cervical stump to optimize the exposure of the stump and allow a safe stumpectomy procedure (Fig. 3).

Excision of the cervical stump using the retrograde colpotomy approach was utilized for the preservation of maximal vaginal length. Then, suturing of the vaginal cuff with a single layer of continuous absorbable sutures was done. She did not require a blood transfusion intraoperatively.

### Postoperative course and follow-up

The patient’s postoperative course was uneventful. Her postoperative hemoglobin was 8.1 g/dl. A transfusion of two units of packed RBCs was performed, and she was discharged on the 5th postoperative day after regular bowel movements were returned and her hemoglobin level was 9.6 g/dl. Histopathological examination of the specimen confirmed the diagnosis of multiple benign leiomyomata of the cervical stump with marked hyaline, mucoid, and cystic degeneration with no atypical features.

## Discussion

One of the simplest and most successful treatments for benign uterine disorders such as fibroids, adenomyosis, and functional uterine bleeding is hysterectomy.

### Route of hysterectomy

Hysterectomies could be performed through minimally invasive approaches such as vaginal or laparoscopic (with or without robotic assistance) approaches or through an abdominal approach. Various factors could influence the selection of a certain route of hysterectomy, such as the size and shape of the uterus and vagina, the degree of extrauterine disease, the need for concurrent procedures, the surgeon’s training and experience, the average case volume, the hospital’s technology, devices, and support, the urgency or scheduling of the case, and the patient’s preference [11]. When possible, minimally invasive hysterectomy techniques (laparoscopic or vaginal, including robot-assisted laparoscopy) should be used due to their established benefits over abdominal hysterectomy.

Among the minimally invasive approaches, ACOG and SOGC recommend the vaginal method, as it was found to be associated with a faster return to normal activities and a better quality of life. Compared with laparoscopic hysterectomy, vaginal hysterectomy is also associated with a shorter operating time and hospital stay [12, 13].

Nevertheless, as per the guidelines and recommendations of the German, Austrian, and Swiss societies of gynecology and obstetrics (DGGG, OEGGG, and SGGG), patients should be given the ability to select the most appropriate therapeutic intervention for their benign uterine disease, considering their individual circumstances [14].

### Total versus subtotal hysterectomy

Based on the extent of the surgery, hysterectomy could be classified into two categories: total hysterectomy (TH), which entails excision of the uterine body and the cervix, and subtotal or supracervical hysterectomy (SH), which describes removal of the uterine body with cervical stump preservation [15].

Before the era of readily available antibiotics and blood transfusions, SCH was much preferred to TH, as it was reported that TH was associated with a significantly higher complication rate, including mortality. At this time, the risk of death for a subtotal hysterectomy performed for fibroids was 1-2.5%, whereas the risk of death for TH performed for benign pathologies including endometriosis and tubo-ovarian abscesses was 3-6.5% [16].

For the following 3 decades, there was a remarkable shift towards total hysterectomies, with the prevalence of SH being approximately 1% of all hysterectomies [6]. The few remaining SH were likely to be conducted when the patient had co-morbidities that necessitated a shorter, less risky procedure, or when the surgeon encountered distorted pelvic anatomy or operative difficulties.

After that, in the late 1990s, subtotal/supracervical hysterectomy regained its popularity, especially due to the introduction of minimally invasive or laparoscopic approaches [17]. It was proposed that this was because SH is simpler to execute during a laparoscopy and because the consensus is that SH would have no or minimal effects on the nerves, vessels, and other pelvic structures, leading to better results in terms of urinary symptoms, pelvic support, and patients’ quality of life [18]. Since then, the merits and drawbacks of subtotal vs. total hysterectomy procedures have been addressed in numerous RCTs, meta-analyses, and Cochrane databases.

Moreover, among the indications of performing a SCH is pelvic organ prolapse (POP). Mesh erosion, a known consequence of mesh sacrocolpopexy following total hysterectomy, could be prevented with laparoscopic sacrocervicopexy, which has been shown to be an effective choice for the management of POP with an excellent safety profile [4, 19, 20].

Despite the historical claims that SH is associated with lower urinary tract complications, better sexual outcomes, and fewer pelvic floor symptoms compared to TH, supracervical hysterectomy has been usually criticized for the high possibility of postoperative long-term sequalae such as cervical dysplasia and carcinoma, cyclic vaginal bleeding, pelvic pain, vaginal discharge, cervical prolapse, and recurrence or de novo development of uterine disorders. Also, there is a significant risk of perioperative bleeding, urinary tract injuries, and gastrointestinal tract injuries in a subsequent extirpation of the cervical stump [10].

### Risk of CIN and cervical carcinoma after SCH

The overall reported risk of cervical stump carcinoma is low; it was reported to be 1–3% of all women who underwent SH, and it represents approximately 3–9% of all cervical malignancies [21]. In a multicenter study on 903 women, only 3 cases (0.33%) of cervical stump carcinoma were observed [22]. It was found that most women undergoing SH are unaware of the recommendations for cervical screening after the hysterectomy [23].

In one survey of women who had minimally invasive hysterectomies, only 67% of women were able to correctly report if the cervix was removed during surgery and the need for future cervical screening as per recommendations [24]. This finding raises the concern that, before offering the option of subtotal hysterectomy, eligible women must be counseled about the importance of continuing their cervical screening program as per guidelines. Furthermore, SH should not be advised for women who have abnormal PAP smears or high-risk HPV infections, as these conditions increase the chance of developing cervical cancer. Historically, it was thought that electrocoagulation of the cervical mucosa during SH might reduce cervical stump carcinoma besides preventing cyclic bleeding after the procedure [25].

SOGC recommends the continuation of routine cervical screening for women with SH as women with an intact uterus. It is also recommended that women with a current or significant history of abnormal cervical cytological smears be informed about the advantages of vaginal or total hysterectomy over SH [26].

### Cyclic vaginal bleeding after SCH

Women undergoing SH are prone to experiencing vaginal bleeding from the retained cervix, which may be persistent and cyclic in some cases. In most cases, this bleeding is minimal, tolerable, and self-limited, especially in well-counselored women [27, 28]. The reported incidence of postoperative bleeding after SH varies between numerous studies, being as low as 0.9% or as high as 25% [29–35], with the majority of studies reporting rates between 5 and 10% [36]. This difference in reported rates might be attributed to the fact that some studies report only cyclic bleeding and others document any form of postoperative bleeding [27]. However, it was found that only half of women undergoing SH were aware that they could suffer from this postoperative bleeding [37].

The available literature is conflicting regarding the risk factors for cyclic bleeding following SH. Heavy menstrual bleeding before hysterectomy was identified as a significant risk factor in one study [37]. While endometriosis was found to be significantly associated with postoperative bleeding in some studies [17, 28–38], others failed to identify a correlation between endometriosis and this bleeding [27].

There are many factors that have been reported to prevent postoperative bleeding after SH, such as older age [37], postmenopausal state [28], and bilateral oophorectomy during the hysterectomy [27]. Moreover, the removal or fulguration of the endocervix during the hysterectomy has been thoroughly studied with inconsistent conclusions [35, 37, 39].

### Recurrence or De novo development of uterine diseases after SCH

Cervical stump leiomyomata, cervical stump cysts, endometriosis, adenomyosis, or cervical stump prolapse are among the comorbidities that may arise or recur in the retained cervix after SH [40]. Additionally, leiomyosarcoma is an extremely rare lesion that may arise in the cervical stump following a SH [41]. It is noteworthy that using power morcellation during laparoscopic supracervical hysterectomy (LSCH) might increase the frequency of recurring fibroids, endometriosis, and adenomyosis [42].

Fibroids have always been described as a “recurring disease.” After myomectomy, there are variable reported rates of recurrence, including 12–15%, 31–43%, 51–62%, and 84% at 1, 3, 5, and 8 years, respectively [43–50], and approximately10–20% of cases will undergo a hysterectomy within 5–10 years of myomectomy [49, 51].

The incidence of fibroids in the retained cervical stump after SCH ranges from 0.6 to 3.7% [9, 52]. In a retrospective analysis of 137 patients who underwent cervical stump resection after SCH, Neis F. et al. reported that 3.7% of these patients have fibroids in their cervical stumps [53]. Similarly, fibroids were found in 2 patients who underwent cervical stumpectomy after SCH in a cohort of 309 patients reported by Hilger et al. [54].

Similar to the case we presented here, recurrence or new development of fibroids over the cervical stump has been described not only after subtotal hysterectomy but also reported after total hysterectomy. Recently, a recurrence of adenomyosis was reported after LSCH with power morcellation [55]. Additionally, benign metastatic leiomyoma (BML) has been described as occurring even after total hysterectomy [56–60].

Among the reported lesions that may arise on top of the retained cervical stump are cervical cysts, which usually result from failure to completely remove all cervical glands. Those cysts are usually benign; however, they may encompass any grade of cervical intraepithelial dysplasia. Initially, cervical cysts may not cause any symptoms. However, persistent cervical cysts may result in a bulging vagina and compression symptoms, including urgency, frequent, and inefficient urination, which may have a major negative impact on the patients’ quality of life. [40, 61].

Table 1. summarizes the reported cases of benign cervical stump lesions reported in the literature. Thirteen cases—in addition to the currently presented case—of histologically proven benign leiomyoma on top of the cervical stump are described. The mean age at presentation is 51 ± 9.36 years, with a mean interval between hysterectomy and symptoms of 7.84 ± 5.8 years. Ten women (71%) had a history of SCH, and four women (29%) had undergone a TH. Preservation of at least one ovary was found in 9/14 (64%) of cases. The indications for the hystectomy were symptomatic fibroids in 10 cases (71%), menorrhagia in 3 cases (21%), and one case of endometriosis (8%). The clinical presentations were pelvic and abdominal pain, vaginal bleeding, and abdominal distention in the majority of cases. One case presented with dysparnia due to a prolapsed fibroid polyp. The mean largest diameter of the excised lesions was 12.4 ± 9.3 cm. Management was achieved via abdominal exploration and excision of the masses with stumpectomy in 9/14 (64%) of the cases; two cases were managed laparoscopically; two cases were managed by vaginal excision of the mass only; and robotically assisted excision of the mass and stumpectomy was performed in one case.

Moreover, cases of malignant leiomyosarcoma, although exceedingly rare, have also been reported after hyetrectomy, with one case from almost 100 years ago, summerized in Table 2 [41, 62, 63].

Nevertheless, patient satisfaction after supracervical hysterectomy was reported to be high [8, 37]. Hence, some patients may elect to have a supracervical hysterectomy even though the evidence shows that there is no clinically significant difference in the rate of complications (such as infection, blood loss, urinary tract, bowel, or vascular injury) and that there is unclear benefit in terms of favorable patient outcomes (such as sexual function, urinary function, or bowel function) between a supracervical hysterectomy and a total hysterectomy. An open abdominal or laparoscopic procedure is most suitable in these circumstances [64]. Therefore, gynecologists should be aware of the advantages and drawbacks of SCH vs. TH, and women requesting a SCH should be properly counseled about the long-term outcomes of the procedure.

**Table 1:** Reported cases of benign cervical stump lesions after supracervical hysterectomy

Author (year)	Patient age (years)	Time to presentation (years)	Hysterectomy Type & indication	Symptoms	Lesion size (cm)	Treatment & pathology
Arthur E. Giles (1923) [65]	46	7	SCH with preservation of one ovary menorrhagia Fibroid uterus	Palpable pelvic mass	Not reported	Abdominal excision Benign leiomyoma
Fuchs IB (2003) [66]	48	5	SCH with BSO Endometriosis	Severe lower abdominal pain & distention	9 × 6.5	Abdominal excision Cystic adenomyoma
Hilger WS [67]	44	5	LSCH with preservation of ovaries Menorrhagia and fibroids	Pelvic pain Pelvic mass	3.2 × 2.7	Robotic assisted resection of the mass
Yanamandra S.R.et al. (2007) [68]	47	8	TAH with preservation of ovaries Menorrhagia	Abdominal pain & brownish vaginal discharge	15 × 10	Abdominal excision Benign leiomyoma
Ismail SM(2009) [69]	47	1	TAH Menorrhagia	Dyspareunia	3 × 3	Vaginal excision Benign leiomyoma
Yarci A (2010) [70]	70	25	TAH with BSO Fibroids	Prolapsed vaginal mass	7 × 3	Vaginal excision Leiomyoma with degeneration
Chu CM (2012) [1]	55	8	SCH with preservation of ovaries Multiple fibroids	Pelvic pain & vaginal bleeding	15 × 9	Abdominal excision Degenerated leiomyoma
	50	8	SCH Symptomatic fibroids	Vaginal bleeding Pelvic pain	20 × 5.7	Abdominal excision Benign leiomyoma
Guraslan H (2015) [71]	62	10	TAH with BSO Symptomatic Fibroids	Abdominal pain & pelvic fullness	20 × 14 × 10	Abdominal excision Cellular leiomyoma
Jayanthi K Krishnamoorthy (2018) [59]	40	6	SCH with preservation of ovaries Fibroid uterus	Abdominal distention	35 × 30 × 25	Abdominal excision after bilateral uterine artery embolization Benign leiomyoma
Mathew and Abraham (2018) [60]	68	Not reported	SCH Fibroid uterus	Lower abdominal pain & distention	Not reported	Abdominal excision Benign leiomyoma
Sezgin B (2021) [72]	50	10	SCH with BSO Menorrhagia & fibroids	Pelvic pain	4	Laparoscopic excision Benign leiomyoma
Tien CT, Ding DC (2023) [55]	47	7	SCH with preservation of ovaries Menorrhagia	Vaginal bleeding	7	Laparoscopic excision Adenomyoma
Present case (2024)	41	2	SCH with preservation of both ovaries Symptomatic fibroids	Pelvic pain, excessive vaginal discharge, bleeding	10.2 × 7.6	Abdominal excision Benign leiomyomata

**Table 2:** Reported cases of malignant leiomyosarcoma after supracervical hysterectomy

Author (year)	Patient age (years)	Time to presentation (years)	Hysterectomy Type & indication	Symptoms	Lesion size (cm)	Treatment & pathology
Barnard (1928) [60]	60	4	SCH Fibroid uterus	Pelvic mass & intestinal obstruction	Not reported	Resection of the mass – patient died 7 h. postoperative
Sturdy (1959) [61]	42	5	SCH with ovarian preservation Fibroid uterus	Pelvic mass	20 × 9 × 7	Radical resection + radiotherapy
Zhiqiang L (2016) [41]	46	3	SCH with ovarian preservation Fibroid uterus	Vaginal bleeding & abdominal pain	6 × 5	Radical resection with lymphadenectomy + chemotherapy + radiotherapy

## Conclusion

Recurrence or De novo development of leiomyomata and other crevical lesions might occur after supracervical or subtotal hysterectomy; thus, thorough pre-operative counseling for women requesting a SCH regarding the pros and cons of the procedure compared with total hysterectomy should be optimized. Meticulous follow-up, including the continuation of routine cervical cytological smears, is mandatory for patients with a retained cervix. We provided a case report of recurrent multiple leiomyomata on top of the cervical stump following a SCH with a comprehensive review of the previously reported cases.

## Data Availability

All data of this manuscript are available upon reasonable request.

## Abbreviations

SCH: Supracervical Hysterectomy
TH: Total Hysterectomy
MRI: Magnetic Resonance Imaging
ACOG: American College of Obstetrics and Gynecology
SOGC: Society of Obstetricians and Gynecologists of Canada
CIN: Cervical Intraepithelial Neoplasia
